# Structural and ligand binding analysis of the pet allergens Can f 1 and Fel d 7

**DOI:** 10.3389/falgy.2023.1133412

**Published:** 2023-03-07

**Authors:** Jungki Min, Alexander C. Y. Foo, Scott A. Gabel, Lalith Perera, Eugene F. DeRose, Anna Pomés, Lars C. Pedersen, Geoffrey A. Mueller

**Affiliations:** ^1^ Genome Integrity and Structural Biology Laboratory, National Institute of Environmental Health Sciences, Durham, NC, United States; ^2^ Basic Research, InBio, Charlottesville, VA, United States

**Keywords:** allergen, dog, cat, lipocalin, structure

## Abstract

**Introduction:**

Pet lipocalins are respiratory allergens with a central hydrophobic ligand-binding cavity called a calyx. Molecules carried in the calyx by allergens are suggested to influence allergenicity, but little is known about the native ligands.

**Methods:**

To provide more information on prospective ligands, we report crystal structures, NMR, molecular dynamics, and florescence studies of a dog lipocalin allergen Can f 1 and its closely related (and cross-reactive) cat allergen Fel d 7.

**Results:**

Structural comparisons with reported lipocalins revealed that Can f 1 and Fel d 7 calyxes are open and positively charged while other dog lipocalin allergens are closed and negatively charged. We screened fatty acids as surrogate ligands, and found that Can f 1 and Fel d 7 bind multiple ligands with preferences for palmitic acid (16:0) among saturated fatty acids and oleic acid (18:1 cis-9) among unsaturated ones. NMR analysis of methyl probes reveals that conformational changes occur upon binding of pinolenic acid inside the calyx. Molecular dynamics simulation shows that the carboxylic group of fatty acids shuttles between two positively charged amino acids inside the Can f 1 and Fel d 7 calyx. Consistent with simulations, the stoichiometry of oleic acid-binding is 2:1 (fatty acid: protein) for Can f 1 and Fel d 7.

**Discussion:**

The results provide valuable insights into the determinants of selectivity and candidate ligands for pet lipocalin allergens Can f 1 and Fel d 7.

## Introduction

The lipocalins are small, ligand-binding, and multi-functional proteins found ubiquitously from bacteria to human (1). Lipocalin family members, in general, share low sequence identity yet high structural similarity with a lipocalin fold consisting of a conserved beta-barrel and an alpha helix. The typical 8-stranded beta-barrel forms a hydrophobic cavity accommodating many small molecules including lipids, vitamins, steroids, and metabolites thus, their functional role is suggested to transport poorly soluble molecules (2). Interestingly, this leads to a diverse array of biological roles that have been proposed including chemical communication, reproduction, regulation of microbiota, innate immunity, and allergy (3–5). Regarding allergy, it has been proposed that the ligands delivered by lipid binding proteins may promote allergic sensitization (6). Hence, identifying the ligand, or ligand class may be useful in understanding their contributions to allergenicity and human health.

The lipocalin family encompasses a number of known allergens - with twenty-four being recorded by in the World Health Organization/International Union of Immunological Societies (WHO/IUIS) Allergen Nomenclature Sub-Committee database (http://www.allergen.org) as of 2022 (Supplementary Table S1) (7). The sources of these allergens are primarily domesticated animals (cow, dog, guinea pig, horse, cat, hamster, and rabbit) and domestic pests like the American and German cockroach. In addition, sensitization to mouse and rat lipocalins as occupational allergens is highly prevalent in laboratory animal handlers (8). All of the lipocalin allergens are respiratory allergens with the exception of Bos d 5, which is a major allergen found in bovine milk associated with common childhood food allergy (9). This near-universal distribution among mammals—especially those which exist in close contact with humans, makes lipocalin allergens a major concern to the scientific community.

Among lipocalins, seven are major allergens (5) which are defined as a single component from the extract to which greater than 50% of patients have specific IgE binding. IgE antibodies to Can f 1 can be found in ∼70% of patients allergic to dog extract (10). Fel d 7 from cats is the most closely related lipocalin and is 63% identical to Can f 1 while the rest of the lipocalins are only 13%–25% identical (Supplementary Figure S1) indicating that these two proteins are from a distinct evolutionary lineage from the other lipocalin allergens (11). Antibodies have been suggested to cross react between Can f 1 and Fel d 7 (12). Cross reactivity is very common when the percent identity is above 70%, and likely above 50% identity (13). Hence, cross reactivity between these two allergens would be expected. However, the prevalence of IgE antibodies to Fel d 7 is only 38%–46% in cat allergic patients (12, 14), suggesting that Can f 1 may be the primary sensitizer.

In the family of lipocalin allergens, crystal structures have been determined for the minor pet allergens Can f 2 (15), Can f 4 (16), and Can f 6 (17), but none of these structures contained a bound ligand that would give insight into the molecule(s) carried by Can f 1 or Fel d 7. However, more distantly related lipocalins have been crystalized with substrates: Bos d 5 with lauric acid (18), Bla g 4 with tyramine (19), Mus m 1 with a pheromone (20), and Rat n 1 with limonene-1,2-epoxide (21). Clearly a diverse array of substrates will bind to lipocalin allergens depending on the protein, however, no lipocalin allergens closely related to Can f 1 or Fel d 7 have been studied. Therefore, in order to understand the possible lipocalin ligands of Can f 1 and Fel d 7, crystal structures were determined, and structure function studies performed to probe the classes of possible ligands. These studies provide new insights into the mechanisms of allergic sensitization by lipocalin allergens.

## Methods

### Protein expression and purification

Codon optimized Can f 1 (Genebank AAC48794, C118S) gene was cloned into pDEST565 having a 6hisGST followed by a TEV cleavage site; C118S was synthesized because free cysteines can sometime lead to problematic expression. Fel d 7 (Genebank ADK56160) gene was cloned into PGEXM vector having a GST followed by a TEV cleavage site. To generate a wild-type Can f 1 gene, site directed mutagenesis was performed using the Quickchange® II (Agilent, CA) with primers; a forward primer 5′-gttcaccctcgcaatacagaatgtagtgatcacgca-3′, and a reverse primer 5′-tgcgtgatcactacattctgtattgcgagggtgaac-3′. For the Can f 1 K131A mutant, a forward primer 5′-tcacgacccagcagcgccgccatacggatctg-3′, and a reverse primer 5′-cagatccgtatggcggcgctgctgggtcgtga-3′, for the Fel d 7 K132A mutant, a forward primer 5′-acgaccaaccagtgcagccatacgcgcctgctc-3′ and a reverse primer 5′-gagcaggcgcgtatggctgcactggttggtcgt-3′ were used. Plasmid DNAs were transformed into *E. coli* BL21(DE3) cells. For protein expression, 30 ml of overnight Luria Bertani culture was transferred into 1 L Terrific Broth media with 100 μg/ml ampicillin and incubated at 37°C until OD 600 nm reached 0.6. Cells were then grown for 1 h at 18°C, induced with 0.5 mM IPTG, and incubated overnight. Cells were harvested by centrifugation at 4,000 g for 15 min. The pellet was resuspended with buffer A (25 mM Tris pH 7.5, 500 mM NaCl) and cells were ruptured by sonication. After removing the cell debris by centrifugation at 47,900 g for 35 min, the supernatant was mixed with 5 ml GST agarose resin in a 50 ml conical tube and incubated on a rocker at 4°C for 30 min with intermittent resuspension. The resin was collected by centrifugation at 500 g for 5 min and washed with 50 ml of buffer A three times. Protein was eluted from the resin with 40 mM glutathione in Buffer A and then cleaved with TEV while dialyzing against Buffer B (25 mM Tris pH 7.5, 150 mM NaCl) overnight. Can f 1 was subsequently passed over Ni-NTA to remove GST prior to gel filtration. The soluble proteins in supernatant were concentrated to 2 ml then passed through a S200 16/60 column equilibrated with buffer B. The peak fraction was collected and analyzed by SDS-PAGE. Protein concentration was calculated using extinction coefficient 0.7378 (mg/ml)^−1^ cm^−1^ for Can f 1 and 1.0735 (mg/ml)^−1^ cm^−1^ for Fel d 7, respectively. The typical yield was 10 mg/L culture. Expression and purification was similar for NMR samples, but the media for expression contained ^13^C and ^15^N isotopes. The proteins used in the NMR studies utilized 1l of M9 media supplemented with ^15^NH^4^Cl, U-^13^C glucose and Celtone base U-^15^N, U-^13^C (CIL). Cells were grown to an OD 600 nm of ∼0.8, temperature was lowered to 18°C, then induced with 1 mM IPTG which was then allowed to express overnight. Pelleted cells were resuspended in 10 ml PBS to which an EDTA-free protease inhibitor cocktail tablet (Roche) and 1 ml B-PER extraction reagent (Thermo Scientific) were added. Cells were sonicated and the resulting supernatant loaded onto a 5 ml GST column. Tagged protein was eluted with PBS containing 50 mM Tris (pH 7.5) and 10 mM reduced glutathione. TEV protease was added to the sample, then cleaved overnight at 4°C. Cleaved protein was then purified using a HiLoad 26/600 Superdex 75pg sizing column (GE).

### Structure determination

Crystallization conditions were screened by sitting drop vapor diffusion method using commercial screens and a Mosquito robot with drops consisting of 200 nl protein (20–26 mg/ml) and 200 nl reservoir solution. Diffraction quality crystals were grown in 35% PEG 4,000 at 4°C for Can f 1 (C118S) and 35% MPD, 100 mM cacodylate pH 6.5, and 0.05 M zinc acetate for Fel d 7 at RT in 48 h. For data collection, crystals were transferred into a cryo solution consisting of the mother liquor and 10%–15% ethylene glycol. Both samples were flash frozen in liquid nitrogen for data collection. Data were collected at APS using SERCAT 22-ID beamline and processed with HKL 2,000 (22). Molecular replacement was performed using Phaser (23) with an Alphafold-predicted structures as a search model (24). The structures were refined with Phenix (25).

### ANS binding assay

Can f 1 and Fel d 7 binding to 8-anilino-1-naphthalene sulfonate (ANS) was assessed using fluorescence measurement with a Polarstar Omega plate reader (BMG Labtech) with 355 nm excitation and 460 nm emission filters. 5 μM of proteins were added to serially diluted ANS in a total reaction volume of 50 μl. The reaction mixture was incubated in 96 well black flat bottom plate (Corning Inc., United States) for 15 min and data were collected at room temperature. Each reaction was performed in triplicate. Another series of measurements without proteins were conducted in the same plate to establish ANS baseline. Data plotting and analysis were performed using Graphpad prism (Graphpad software, CA).

### Fatty acid screening

Can f 1 and Fel d 7 binding to ligands were assessed using ANS displacement assay with fatty acid screening library (Cayman chemical, MI) (26). Final 0.2 mg/ml of proteins were premixed with 2 μM ANS. Then, 10 μM fatty acids were added into the reaction mixture in a total volume of 50 μl. The reaction mixture was incubated in 96 well black flat bottom plate (Corning Inc., United States) for 1 h at RT. Data were collected at room temperature with a Polarstar Omega plate reader (BMG Labtech) with 355 nm excitation and 460 nm emission filters. Each reaction was performed in triplicate. The change in fluorescence signal (F) was normalized by using the following equation; % fluorescence reduction = 100−(F_fatty acid_−F_ANS alone_)/(F_no fatty acid_−F_ANS alone_) × 100.

### Circular dichroism measurement

CD measurements were performed using a J-1500 spectropolarimeter (Jasco, Tokyo, Japan) at RT. Five µM proteins in PBS were transferred into a 10 mm quartz cuvette and scanned from 200 nm to 260 nm with 4 times repetition. The baseline measurement was performed to subtract from each spectrum. The data are expressed as molar residue ellipticity (θ). Thermal melting experiment was conducted using the same sample used for CD measurement and a PTC-517 Peltier with increasing temperature from 22°C to 95°C. Each measurement was performed in triplicate.

### NMR experiment

Carbon-proton HSQC correlated spectroscopy was used to assess the chemical shifts before and after adding 200 μM pinolenic acid to a buffer of PBS with 100 μM protein, either Can f 1 or Fel d 7. The NMR sample was concentrated to ∼300 μM in a buffer consisting of PBS, 0.25 mM sodium azide, 10% D_2_O pH 7.5. Pinolenic acid was loaded by diluting the protein sample to 4 ml, then adding a 4X concentration of pinolenate. Sample was heated to 95°C for 30 min, then slowly cooled. It was washed 3X, then re-concentrated to ∼300 μM. An Agilent 800 MHz spectrometer with a cryogenically cooled probe was used.

### Docking and molecular dynamics simulation

Using Autodock-vina (27), oleic acid (Ole), prostaglandin H2 (PH2), pinolenic acid (PIN), and retinoic acid (RAC) ligands were docked onto Can f 1 and Fel d 7 crystal structures and the docked conformations were the starting configurations for the molecular dynamics trajectories. Protons were introduced to the proteins by using the leap module of Amber.18 (28). Na^+^ and Cl^−^ ions were added to provide the 100 mM effective ionic concentration, plus an additional four and five Na^+^ ions for charge neutralization of Can f 1 and Fel d 7 systems, respectively. The system was solvated in a box of TIP3P water with the box boundary extending to 20 Å from the nearest peptide atom. All Lys, Arg, Glu and Asp residues were in their charged states. His113 of Can f 1 and His114 of Fel d 7 were considered δ-protonated while remaining His residues of Can f 1were deemed ε-protonated. Prior to equilibration, the solvated system was sequentially subjected to (1) 500 ps belly dynamics with fixed peptide, (2) minimization (5,000 steps), (3) constant temperature (300 K)-constant pressure (1 atm) dynamics (∼1 ns) at fixed protein to assure a reasonable starting density around 1 g/cc, (4) minimization (5,000 steps), (5) stepwise heating MD at constant volume (to bring the temperature up to 300 K in 3 ns), and (6) constant volume simulation for 10 ns with a constraint force constant of 10 kcal/mol applied only on backbone heavy atoms. After releasing all constraining forces within the next 20 ns of the equilibration period, MD trajectories were continued at constant temperature (Langevin thermostat) constant volume molecular dynamics simulations for 3 μs each (Supplementary Figure S2). All trajectories were calculated using the PMEMD module of Amber.18 with 1 fs time step. Long range coulombic interactions were handled using the PME method with a 10 Å cut-off for the direct interactions. The amino acid parameters were selected from the FF14SB forcefield of Amber.18, the ligand forcefield was selected from the gaff2 parameters in Amber.18, and the partial atomic charges were generated from single point B3LYP/6-31 G* calculations of an optimized geometry using Gaussian-16 (29). The Amber parameter files for the ligands in the form of Amber library files can be made available upon request. At the salt concentration of 100 mM, the MMGBSA module with the standard parameters was used to estimate binding energies from 1,000 samples selected from molecular dynamics simulations for each ligand.

### Stoichiometry determination

Can f 1 and Fel d 7 were mixed with saturating amounts of ^13^C-methyl-oleic acid and annealed to 80°C. Upon cooling to room temperature, excess lipid was removed by centrifugation, followed by filtration, and then 3 buffer exchanges to PBS. The protein was then mixed 1:1 with 10% w/v cholate to dissolve the lipids for accurate quantitation of oleate. A ^13^C-methyl-oleate standard curve of the methyl intensity of the ligand in a series of HSQC spectra with known concentrations (also diluted in 1:1 10% w/v cholate) was prepared to compared intensities with the amount of oleate extracted by the protein.

## Results

### Comparison of Can f 1 and Fel d 7 structures

**Figure 1:**
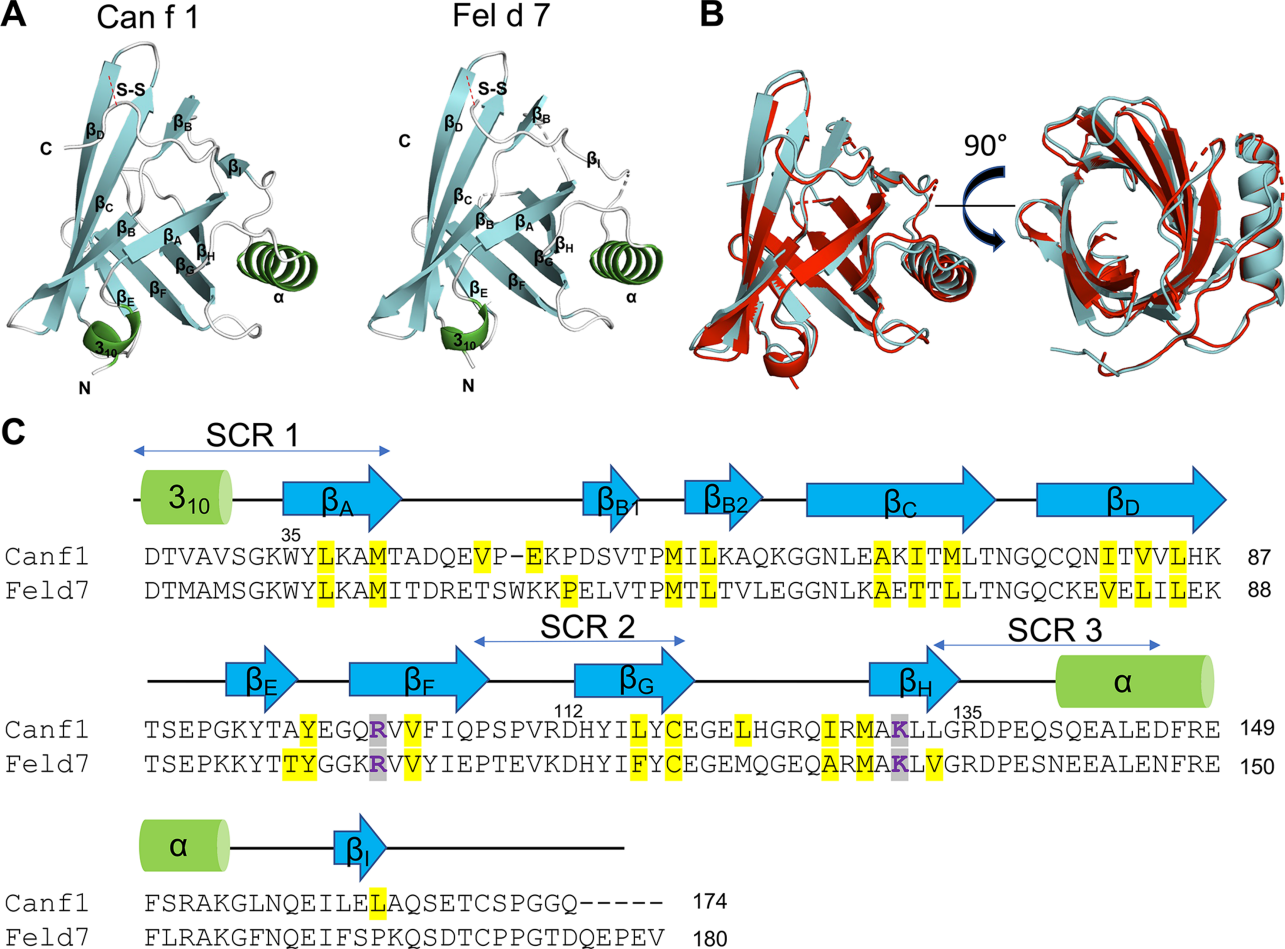
Structures of Can f 1 and Fel d 7. (**A**) Structure of Can f 1 (C118S) (left) and WT Fel d 7 (right). (**B**) Can f 1 (C118S) and WT Fel d 7 were superimposed with RMSD value of 1.0 Å. (**C**) Sequence alignment of Can f 1 and Fel d 7. Key charged residues in the calyx R101 and K131 for Can f 1, and R102 and K132 for Fel d 7 are colored purple. Other residues lining the calyx are highlighted in yellow. Secondary elements are depicted as green cylinder for *α*-helix and blue arrows for β-strands.

**Figure 2:**
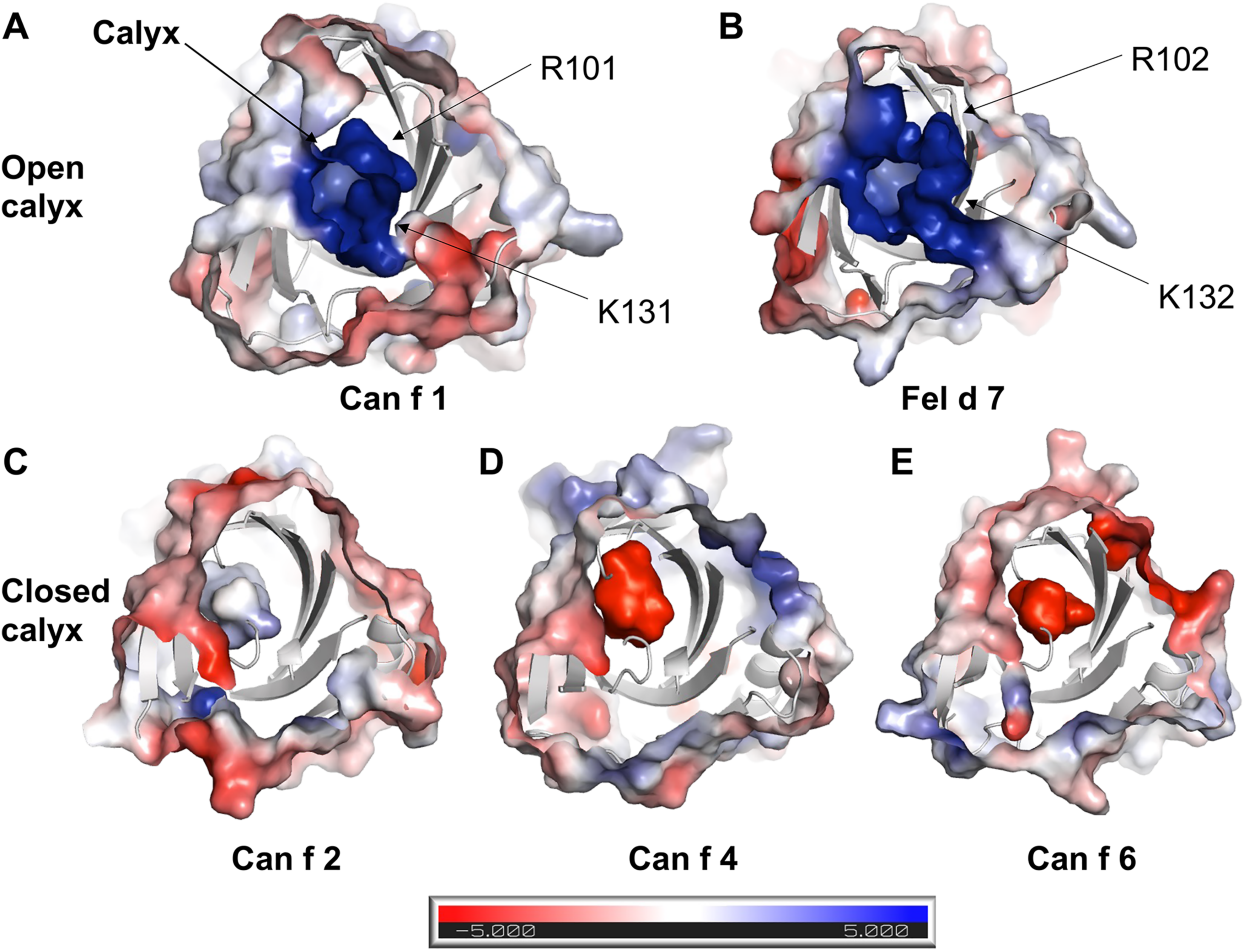
Electrostatic surface of lipocalins. Electrostatic surface of (**A**) Can f 1, (**B**) Fel d 7, (**C**) Can f 2, (**D**) Can f 4, (**E**) Can f 6 are calculated using ABPS module in PyMol. The central calyx is shown with the arrow in A. Can f 1 and Fel d 7 show an open calyx while Can f 2, Can f 4, and Can f 6 show closed calyxes.

To obtain molecular insights into how lipocalins function as allergens, we determined crystal structures of a major dog allergen Can f 1 (C118S, aa 27–171) and its cross-reactive homolog minor cat allergen Fel d 7 (wild-type, aa 27–170) at a resolution of 1.6 Å and 2.2 Å, respectively (Table 1). Recently, a Can f 1 (C118A) crystal structure was reported at a resolution of 2.5 Å (PDBID:7DRU) (30). Our Can f 1 (C118S) structure, hereafter Can f 1, has a higher resolution and contains one molecule per asymmetric unit. It superimposes with the Chain C of 7DRU with an RMSD value of 0.73 Å for 814 atoms, and shows more ordered side chains; therefore, we will use our Can f 1 structure for analyses in this manuscript. Can f 1 and Fel d 7 have a typical lipocalin fold consisting of central eight anti-parallel beta strands, a flanking 3_10,_ and an α-helix close to the C-termini (Figure 1A). They superimpose with an RMSD value of 1.0 Å for 1,412 atoms (Figure 1B) and share 63% sequence identity (Figure 1C), suggesting close functional similarity. While Can f 1 shows electron densities for most loops flanking beta-strands, some Fel d 7 loop regions are disordered: Arg44-Trp48, Glu122-Gln127, and Asn158-Glu160. The most disordered regions for both structures are the N- and C-termini. Both Can f 1 and Fel d 7 have an open positively charged calyx at the center of an anti-parallel beta-barrel (Figures 1C, 2). While the calyx of Can f 1 is somewhat larger than that of Fel d 7, both have significantly more interior surface area and volume than other dog lipocalins such as Can f 2 (15), Can f 4 (16), and Can f 6 (17). Closer examination of the calyx surface reveals a concentration of positively-charged residues. This appears to be unique among other lipocalins, which have a neutral or negatively-charged character further reinforcing the structural/functional similarities between Can f 1 and Fel d 7 (Table 2 and Figure 2). Unfortunately, we observed no ligand-like electron density in the calyx of either allergen that might provide clues as to the class of ligands that might bind there.

**Table 1:** Selected crystallographic data.

	Can f 1 (C118S)	Fel d 7
	PDB ID: 8EPU	PDB ID: 8EPV
**Data collection**		
Space group	C 1 2 1	P 4_1_ 2_1_ 2
Cell dimensions		
a, b, c (Å)	62.2, 36.5, 58.0	85.9, 85.9, 40.1
α, β, γ (°)	90, 100.8, 90	90, 90, 90
Wavelength (Å)	1.0	1.0
X-ray Source	APS SER-CAT 22ID	APS SER-CAT 22ID
Resolution (Å)^a^	50.0–1.60 (1.63–1.60)	50.0–2.20 (2.24–2.20)
No. of Reflections [unique]	16,064 [5,182]	59,748 [8,074]
Completeness (%)	94.0 (78.7)	99.5 (93.3)
*R_merge_*^a^^,b^ (%)	4.6 (18.6)	8.8 (79.0)
*I*/*σ*^a^	26.0 (3.0)	22.4 (1.5)
Redundancy^a^	3.1 (2.5)	7.4 (4.7)
**Refinement**		
Resolution (Å)^a^	31.3–1.60 (1.70–1.60)	38.4–2.19 (2.30–2.20)
Molecules per AU	1	1
No. of a.a/AU	137	144
No. of waters/AU	68	31
*R_work_*^c^ (%)	21.1	24.1
*R_free_*^d^ (%)	23.0	26.5
Average *B*-factors (Å^2^)		
Protein	36.3	56.6
Water	38.9	51.0
R.m.s. deviations		
Bond lengths (Å)	0.009	0.005
Bond angles (°)	1.020	0.722
Ramachandran (%)		
Favored, Allowed, Outlier	98.60, 1.40, 0	99.23, 0.77, 0

^a^ Values in parenthesis are for highest-resolution shell.

**Table 2:** Comparison of calyx.

Allergen (PDB ID)	Cavity Surface Area (Å^2^)^a^	Cavity Volume (Å^3^)^a^
Can f 1 (8EPU)	466.9	274.6
Fel d 7 (8EPV)	260.2	131.6
Can f 2 (3L4R)	185.5	97.7
Can f 4 (4ODD)	158.3	95.0
Can f 6 (5X7Y)	127.8	52.5
LCN1^b^ (1XKI)	482.5	394.3
Fel d 4^c^	118.6	59.1

^a^ Cavity surface area and volume are calculated using CASTp (http://sts.bioe.uic.edu/castp/index.html?3igg).

^b^ Human Tear Lipocalin (31).

^c^ Modeled using AlphaFold (24).

### Ligand screening

**Figure 3:**
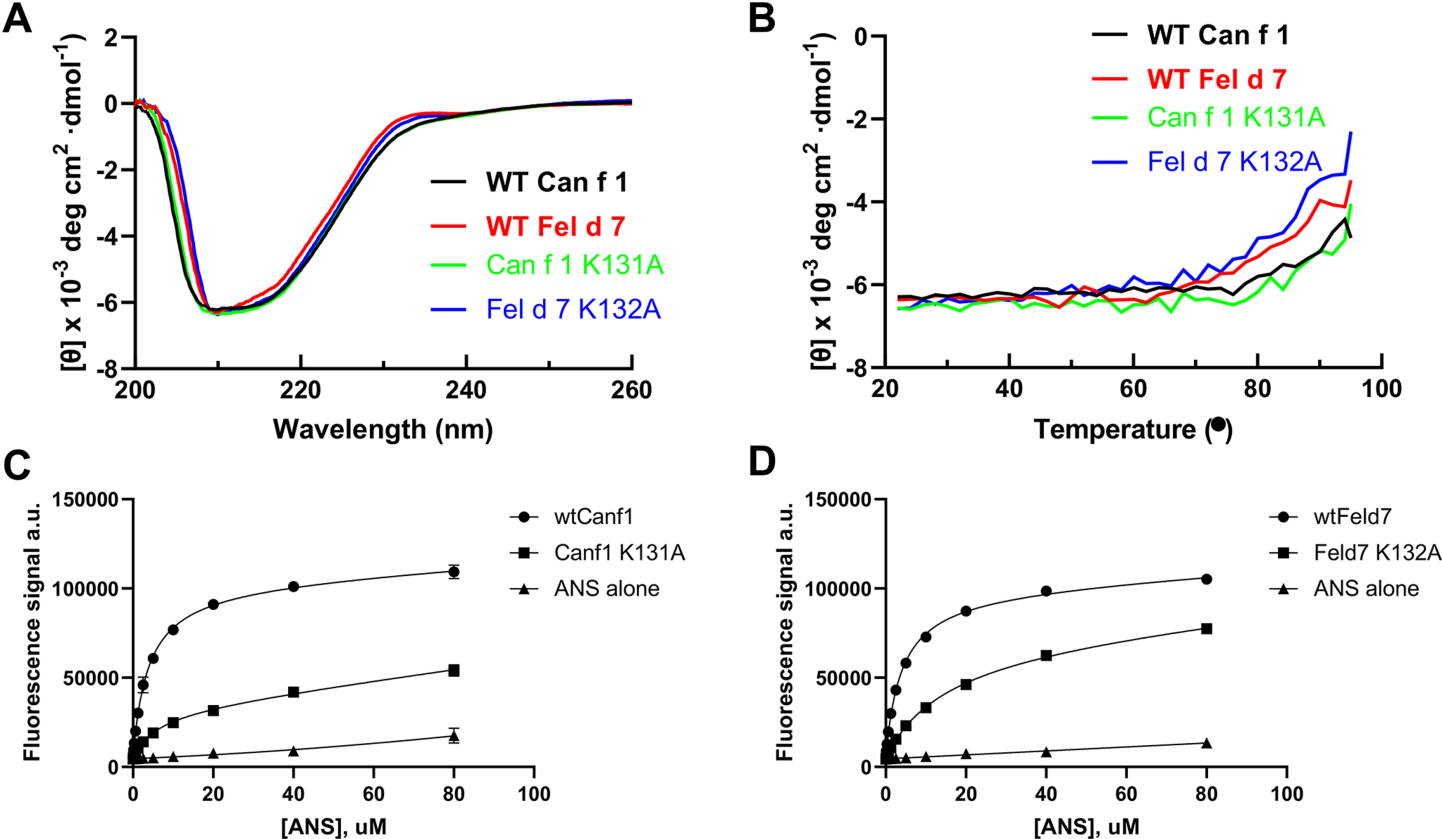
ANS binding to Can f 1 and Fel d 7. (**A**) The far-UV CD spectra of the wild-type and mutant proteins are shown. (**B**) The CD melting curve of wild-type and mutant proteins are measured at 210 nm in PBS buffer pH 7.4. The fluorescence increase upon ANS titration is shown for (**C**) WT Can f 1, Can f 1 (K131A), (**D**) WT Fel d 7, and Fel d 7 (K132A).

This open positively charged calyx is a novel feature among lipocalins, potentially impacting the ligand binding selectivity of Can f 1 and Fel d 7. To explore this hypothesis, we employed an ANS (8-Anilino-1-naphthalene sulfonic acid)-based displacement assay. Binding of ANS to a hydrophobic cavity such as the calyx of Can f 1/Fel d 7 significantly increases its fluorescence intensity, which can be measured to yield a binding curve (26). This approach has been used to study other lipocalins previously (32). To verify its use here, we examined the x-ray structure and identified a potential salt bridge between the carboxylate of ANS and K131 in Can f 1 and K132 in Fel d 7. K131A (Can f 1) and K132A (Fel d 7) mutants were created to eliminate this salt bridge. Loss of the salt bridge did not significantly alter protein folding or stability when assessed using Circular Dichroism (CD) (Figures 3A,B). Despite this, the alanine mutants displayed significantly reduced ANS binding, with binding lower maximum fluorescence values and higher Kd's when compared to the binding curves of the wild-type proteins. (Figures 3C,D). This mutational analysis suggested that ANS binds in the calyx.

**Figure 4:**
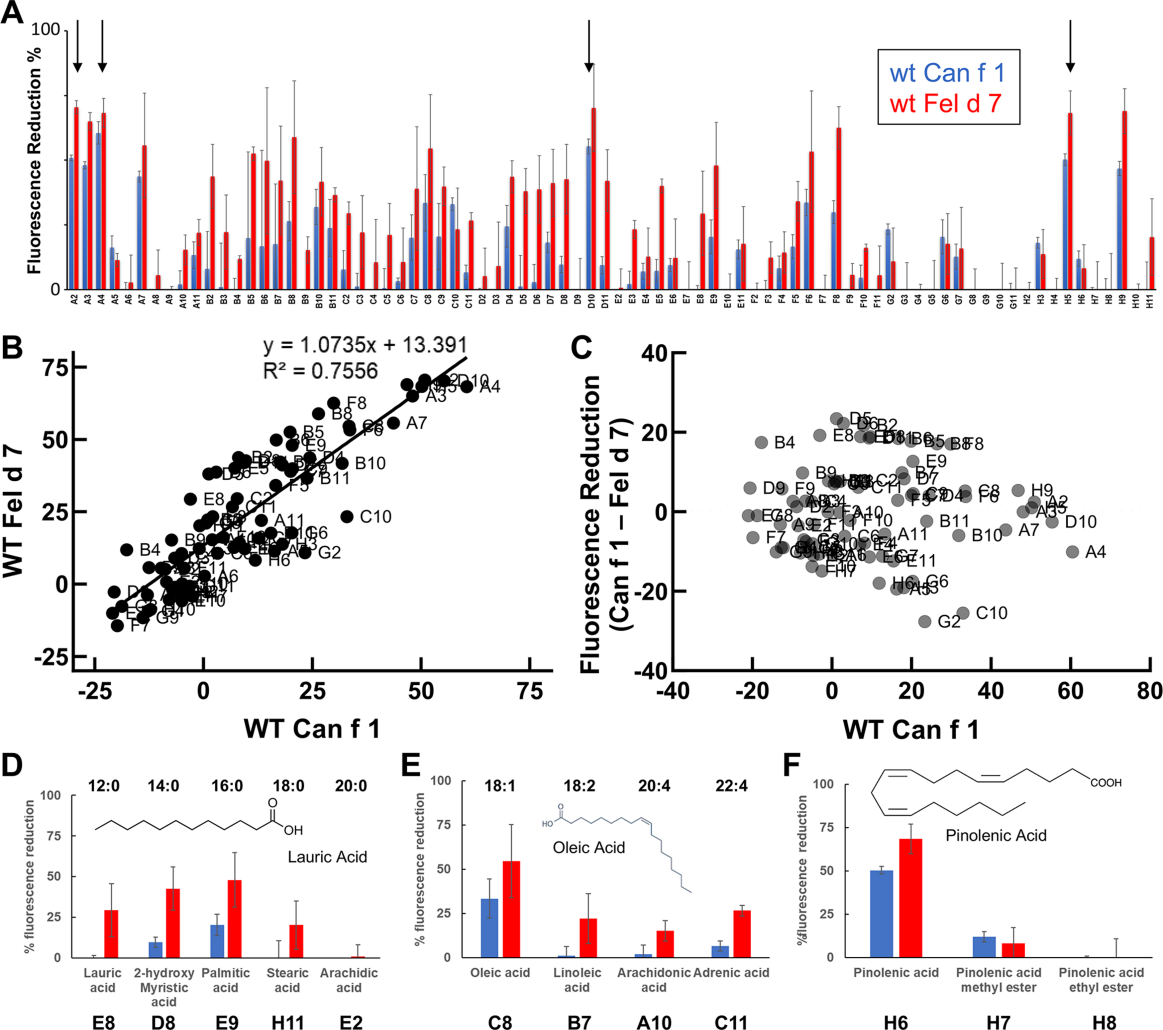
Can f 1 and Fel d 7 ligands. (**A**) WT Can f 1 and WT Fel d 7 are screened against a fatty acid library using ANS-displacement assay. The % reduction of fluorescence in the presence of 1 µM fatty acids is shown in blue for WT Can f 1 and red for WT Fel d 7 ligands, respectively. All measurements are duplicated, and the error bar indicate standard deviation. The labels on the x-axis correspond to the ligands in Supplementary Table S2. Arrows indicate A2, A4, D10, and H5. (**B**) The correlation plot shows a linear relationship between Can f 1 and Fel d 7 ligands. (**C**) The residual plot shows the difference between Can f 1 and Fel d 7 displacement for each ligand. (**D**) The % fluorescence reduction in the presence of the saturated fatty acids of certain carbon length. Note that 16 carbon fatty acid shows the largest % fluorescence reduction, suggesting the greatest binding capability. (**E**) The unsaturated fatty acids binding to WT Can f 1 and WT Fel d 7 is shown with the carbon length (first number) and the position of double bond (second number). (**F**) Further analysis shows the % fluorescence reduction for pinolenic acid with/without ester modification. Bar and whiskers plots indicate error bars.

Many allergens bind lipids (33); therefore, we selected fatty acids as a surrogate group for screening. Having reasonably established that ANS binds to the calyx, we utilized a fluorescence-based competition assay whereby the displacement of ANS would reduce the fluorescence signal when other ligands bound. We screened a total of 75 fatty acids binding to wild-type Can f 1 and Fel d 7. In general, Can f 1 and Fel d 7 show a similar pattern of percent fluorescence reduction to each fatty acids (Figure 4A). Among the screened fatty acids (Table 2), N-(α-Linolenoyl) tyrosine (A2), N-Oleoyl-L-serine (A4), Linoleoyl glycine (D10), and Pinolenic acid (H5) showed greater than 50% fluorescence reduction for both Can f 1 and Fel d 7, suggesting relatively higher binding capability (Black Arrows, Figure 4). Can f 1 (C118S) and wild type Can f 1 showed similar pattern of the percent fluorescence reduction (data not shown). The similarities and differences for each ligand are plotted in the correlation and residual plots (Figures 4B,C). The results suggest that Can f 1 and Fel d 7 ligands are highly correlated, and outliers are scarce. Looking for patterns in the data, a chain length for saturated lipids around C16–18 appears optimal for binding (Figure 4D). Although, panel E shows that adding a double bond to stearic acid (18:0), results in improved binding by oleic acid (18:1). However, this pattern does not extend to saturated chains for arachidonic acid. The selection for a negatively charged ligand is exemplified by the high ANS displacement signal for pinolenic acid that is substantially reduced when the carboxylate is replaced by a methyl or ethyl ester (Figure 4F). This is consistent with the positively charged nature of the Can f 1 and Fel d 7 calyx.

### NMR validation of ligand binding

**Figure 5:**
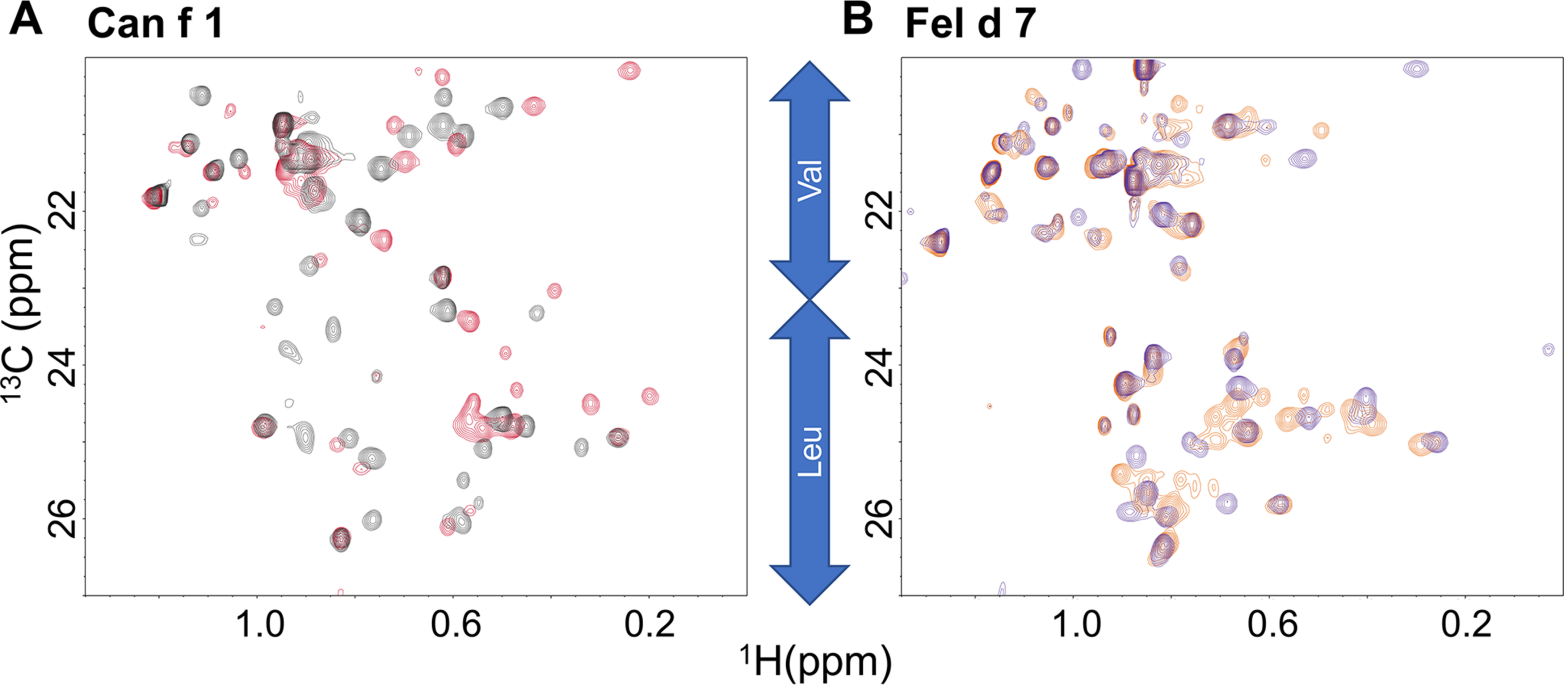
Heteronuclear single quantum coherence (HSQC) spectra. The HSQC spectra of Can f 1 (**A**) and Fel d 7 (**B**) show chemical shift perturbations for hydrophobic residues in the absence (red) and presence (black) of 200 µM pinolenic acid for WT Can f 1. The similar trend is shown in the absence (orange) and presence (purple) of 200 µM pinolenic acid for the WT Fel d 7.

While the ANS mutational analysis in Figure 3 suggested that ANS binds in the calyx, NMR was used to validate that pinolenic acid binds in the open calyx. The ^13^C Heteronuclear Single Quantum Coherence (HSQC) spectra focusing in on the Leu/Val region shows that the addition of pinolenic acid to Can f 1 and Fel d 7 results in substantial chemical shift changes (Figure 5). In both spectra, Valine and Leucine methyl group peaks are widespread and most of the changes are below 0.8 ppm in the ^1^H dimension, indicating that conformational changes occurred when the long chain of the pinolenic acid interacted with many different residues in the interior of proteins. The sharper methyl peaks around 0.8 ppm, likely to be exterior residues, changed the least. These results support our hypothesis that ligand binds inside the calyx.

### Ligand docking and molecular dynamics simulation

**Figure 6:**
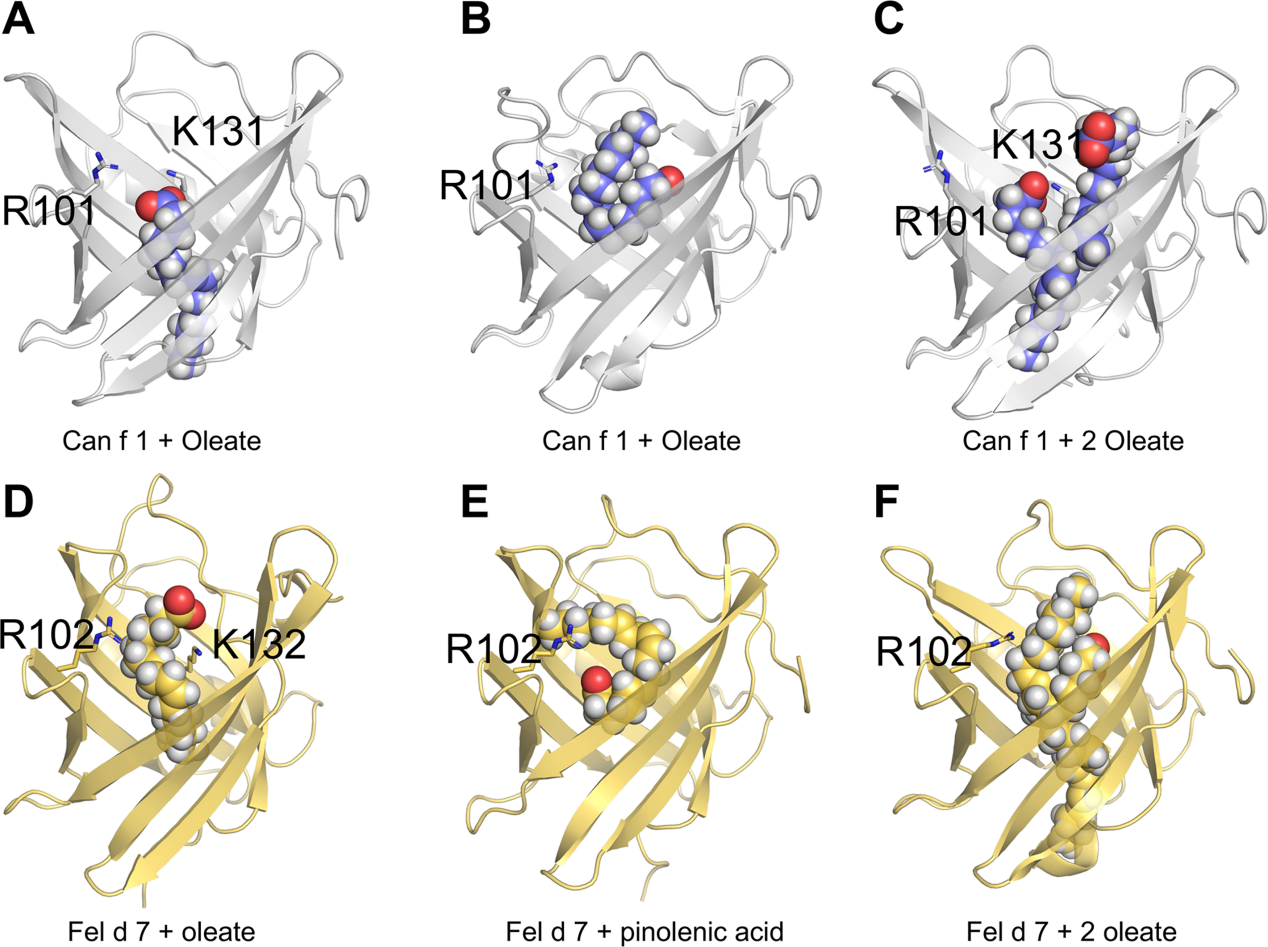
Molecular modeling of Can f 1 and Fel d 7. Models of Can f 1 with oleate (**A & B**), and 2 oleate molecules (**C**). Models of Fel d 7 with oleate (**D**), pinolenic acid (**E**), and 2 oleate molecules (**F**). Ligands are shown as sphere models, and key side chains of residues R101 and K 131 for Can f 1, and R102 and K132 for Fel d 7 are rendered as sticks.

To understand how fatty acids interact with calyx, we attempted co-crystallization of Can f 1 and Fel d 7 with ANS or pinolenate. Unfortunately, we obtained either no crystals or poor quality data. We also performed docking simulations with AUTODOCK using oleate and pinolenate as representing ligands for Can f 1 and Fel d 7. Initially, we defined the ligand-binding position to be inside the calyx. Then, after docking simulation, we picked reasonable models based on the negative free energy and proximity to the positively charged lysine. The selected docking model shows that oleate fits into the calyx with the acyl tail pointing inwards in Can f 1 (Figure 6A) and Fel d 7 (Figure 6D).

Subsequent molecular dynamics simulations for 3 microseconds showed that the carboxylate of the ligands samples between the two positively charged residues (R101 and K131 for Can f 1; R102 and K132 for Fel d 7). During the course of the simulation the acyl chain of oleate would occasionally lay superficial to the calyx as shown in the example with Can f 1 (Figure 6B). Similarly in simulations with Fel d 7 using oleate and pinolenic acid, a similar positioning was noticed (Figure 6E). Moreover, the acyl tail orientation and the interactions with another positive residue suggested that an extra lipid molecule would fit simultaneously into the calyx. Indeed, simulations with 2 oleate molecules confirmed that 2 oleates could remain bound for up to 3 μs to Can f 1, however 2 oleate molecules only stayed briefly bound to Fel d 7. Examples from the simulation with 2 oleate molecules bound to the lipocalins are shown in Figures 6C,F. Supplementary Table S3 shows calculated affinities during MD simulations for various ligands with variable numbers of carbons and double bonds including oleate, pinolenate, prostaglandin H2, and retinoic acid. For both lipocalins similar affinities were calculated for all the ligands highlighting the promiscuous binding of negatively charged lipids with similar sizes and variable saturation.

### NMR stoichiometry measurement

To confirm the hypothesis that 2 oleates can bind the calyx simultaneously, we measured the stoichiometry of oleate binding to Can f 1 and Fel d 7. Using methyl-^13^C-oleate, we created a standard intensity curve using the methyl signal of the ligand in a series of NMR spectra. The intensity was compared with the amount of methyl-^13^C-oleate that would anneal with either protein, after removing excess soluble oleate. For both Can f 1 and Fel d 7, approximately a 2:1 ratio of oleate to protein was measured. (Supplementary Figure S3). The data are consistent with the simulations and our biochemical and structural data, suggesting how Can f 1 and Fel d 7 ligands bind to the calyx.

## Discussion

Dogs and cats are important sources of indoor allergens affecting 10%–20% of the population (34). We determined crystal structures of related lipocalin allergens Can f 1 and Fel d 7 to provide insights into the role of ligands (Figure 1). Surface charge analysis revealed that Can f 1 and Fel d 7 have an open positively charged hydrophobic calyx. This is unique among lipocalin structures studied to date, potentially influencing the chemical nature of their preferred ligands (Figure 2). Based on this conjecture, we screened and identified candidate ligands for recombinant Can f 1 and Fel d 7 using fatty acids as surrogate lipids (Figure 4). Here both Can f 1 and Fel d 7 showed a preference for 16–18 carbon saturated and polyunsaturated fatty acids. The negative charge of the acid head group is neutralized by the positively charged environment of the calyx, either by R101/K131 or R102/K132 in Can f 1 or Fel d 7, respectively. The MD simulations confirm that the positively charged residues in the calyx play a key role in accommodating the negative charge of ligands, ANS binding to mutants, and reduced competition of ester derivatives. Therefore, we propose that these classes of ligands, namely C16–18 carbon saturated and polyunsaturated fatty acids, represent close approximations of the natural ligands for these lipocalins. However, there is significant promiscuity indicating that many fatty acid ligands will bind.

We were unable to see melting temperature change and proteolytic degradation of Can f 1 and Fel d 7 in the absence and presence of fatty acids (data not shown) likely due to the high intrinsic stability which has been noted for other lipocalins (35, 36). In other allergens like Bla g 1, the bound ligand can further stabilize these proteins potentially affecting antigen processing (37, 38). Due to the high thermal stability and proteolytic resistance, the experimental validation of a stabilizing effect due to the ligand was challenging. However, the *in-silico* analysis suggests that stable conformations of candidate ligands can be achieved *via* interactions with calyx residues. While Can f 2, Can f 4 and Can f 6 have neutral to negatively charged closed calyx, Can f 1 and Fel d 7 display an open calyx, which appears to support up to two fatty acids based on molecular modeling and NMR titrations (Figures 6C,F and Supplementary Figure S3).

Allergens are not universally distributed among all protein folds (39). In fact, some folds are quite common including lipocalins. In a survey of other ligand binding allergens (6) it was noted that fatty acids, like those proposed to interact with Can f 1 and Fel d 7, bind to the PR-10 family (40), non-specific lipid transfer proteins (nsLTP) (41), serum albumins (SA) (42), Major Allergen (MA) domains such as Bla g 1 (38), and uteroglobulins (43). How these fatty acids may influence the immune system is still an active area of investigation. Aeroallergens encounter two lipid-based barriers when inhaled. These are the pulmonary surfactant and the luminal plasma membrane of airway epithelial cells. The exchange or delivery of various lipids may affect the stability of these membrane structures. For example, the MA domains AEG12 and Bla g 1 lyse mammalian cells and AEG12 disrupts replication of enveloped viruses (44, 45). The nsLTP Ole e 7 has a similar high affinity to MA domains for charged phospholipids and it was shown to absorb to air-liquid interfaces. It is possible that many of these lipids and/or the lipid binding proteins function as detergents disrupting natural membranes or the surface tension of air-liquid interfaces. One of the current major hypotheses about the rise of allergy and autoimmunity in the modern era is that it is due to epithelial barrier disruptions (46). While more work will need to be done to prove a cause and effect with lipid binding allergens, this study provides a feasible biochemical mechanism whereby allergens with a moderate affinity for fatty acids may unload their cargo into mucosal surfaces disrupting normal epithelial barriers.

In conclusion, we propose candidate fatty acids as ligands of Can f 1 and its related Fel d 7. We identified that the Lys131 of Can f 1 (Lys 132 for Fel d 7) in the calyx is the critical residue for ligand binding. Future study is needed to address how these ligands affect allergy sensitization in cellular and animal models.

## Data Availability

The datasets presented in this study can be found in online repositories. The names of the repository/repositories and accession number(s) can be found below: http://www.wwpdb.org/, 8EPU for Can f 1, 8EPV for Fel d 7.
