# Supplementary material for: Structural and ligand binding analysis of the pet allergens Can f 1 and Fel d 7
Source: Front Allergy. 2023 Mar 7;4:1133412. doi: 10.3389/falgy.2023.1133412 (PMC10028261; doi:10.3389/falgy.2023.1133412)
Supplement: Supplementary file 1 [file Presentation1.pdf]

Supplementary Figure 1, Identity Matrix of Lipocalin Allergens

	Bla g 4	*Per a 4*	*Can f 1*	*Fel d 7*	*Can f 2*	*Mus m 1*	*Rat n 1*	*Cav p 6*	*Ory c 4*	*Equ c 1*	*Can f 6*	*Fel d 4*	*Bos d 2*	*Equ c 2*	*Can f 4*	*Ory c 2*	*Phod s 1*	*Mes a 1*	*Cav p 3*	*Cav p 1*	*Cav p 2*
Bla g 4	100	24	13	15	17	14	16	18	11	14	14	16	13	15	9	16	14	12	13	12	18
Per a 4	24	100	15	13	15	12	19	17	11	15	13	18	12	15	10	13	11	13	12	13	17
Can f 1	13	15	100	63	21	18	20	21	22	25	22	25	16	16	16	22	19	19	18	18	20
Fel d 7	15	13	63	100	22	21	23	23	23	24	24	22	16	20	18	19	19	17	20	19	21
Can f 2	17	15	21	22	100	26	29	27	29	26	25	25	17	21	22	20	13	18	20	18	14
Mus m 1	14	12	18	21	26	100	67	45	49	48	49	52	24	27	26	26	22	26	26	29	29
Rat n 1	16	19	20	23	29	67	100	49	52	50	54	58	27	34	29	30	25	28	27	31	32
Cav p 6	18	17	21	23	27	45	49	100	49	50	54	55	25	25	23	27	25	25	23	26	24
Ory c 4	11	11	22	23	29	49	52	49	100	52	56	60	27	28	31	21	22	23	23	27	30
Equ c 1	14	15	25	24	26	48	50	50	52	100	60	70	31	34	25	25	22	32	28	29	32
Can f 6	14	13	22	24	25	49	54	54	56	60	100	70	27	34	26	23	28	31	27	29	28
Fel d 4	16	18	25	22	25	52	58	55	60	70	70	100	31	35	25	25	23	31	28	31	33
Bos d 2	13	12	16	16	17	24	27	25	27	31	27	31	100	34	32	33	33	31	38	37	39
Equ c 2	15	15	16	20	21	27	34	25	28	34	34	35	34	100	31	27	32	34	33	33	36
Can f 4	9	10	16	18	22	26	29	23	31	25	26	25	32	31	100	30	29	31	31	33	32
Ory c 2	16	13	22	19	20	26	30	27	21	25	23	25	33	27	30	100	35	41	33	34	39
Phod s 1	14	11	19	19	13	22	25	25	22	22	28	23	33	32	29	35	100	39	40	39	39
Mes a 1	12	13	19	17	18	26	28	25	23	32	31	31	31	34	31	41	39	100	40	42	38
Cav p 3	13	12	18	20	20	26	27	23	23	28	27	28	38	33	31	33	40	40	100	46	46
Cav p 1	12	13	18	19	18	29	31	26	27	29	29	31	37	33	33	34	39	42	46	100	49
Cav p 2	18	17	20	21	14	29	32	24	30	32	28	33	39	36	32	39	39	38	46	49	100

Supplementary Figure 2

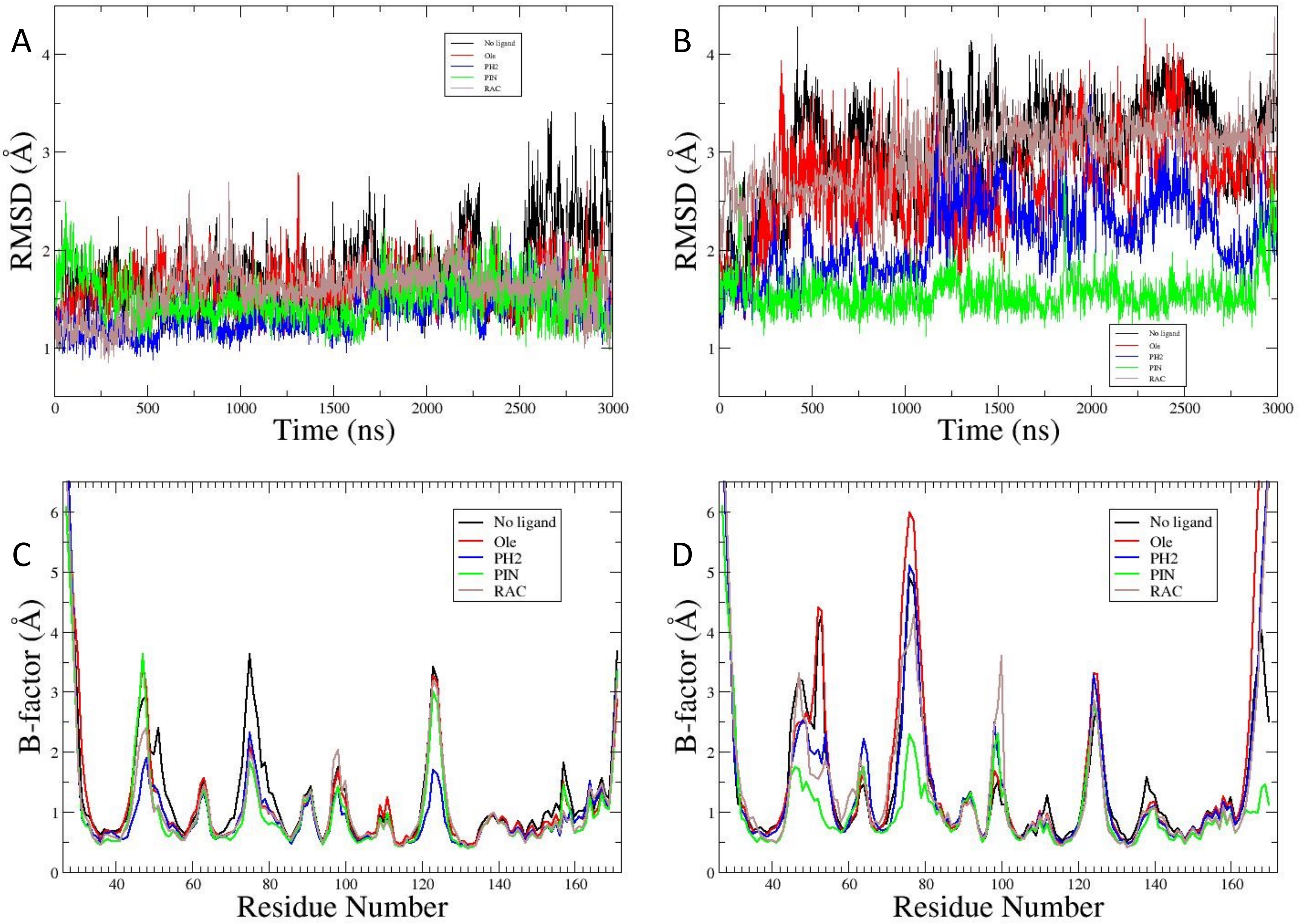

Dynamic Characterization of Ligand Bound Protein Structures. Root mean squared deviations are shown over time for backbone atoms during the MD simulation of A) Can f 1 and B) Fel d 7. The thermal fluctuations over time of individual residues were converted into B-factors for C) Can f 1, and D) Fel d 7. The colors correspond to simulations with different ligands: Black none, Red oleate (OLE), Blue prostaglandin H 2 (PL2), Green pinolenic acid (PIN), and Purple all-trans-retinoic acid (RAC). In nearly all cases Fel d 7 shows increased fluctuations compared to Can f 1.

Supplementary figure 3

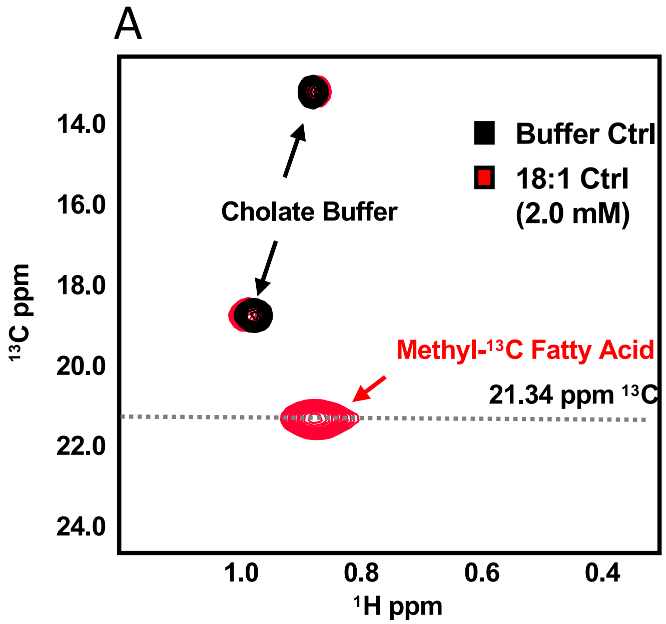

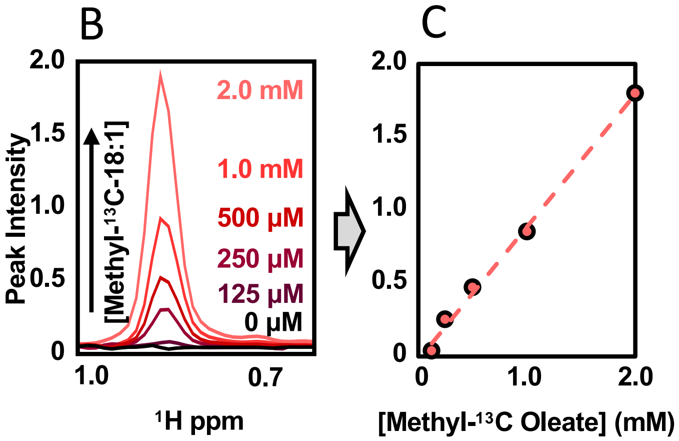

D

	Can f 1	Fel d 7
**Mol. Ratio**	1.8 ± 0.3	2.3 ± 0.4

Stoichiometry Measurements by NMR. A) 2D HSQC of ^{13}C methyl oleate in cholate buffer. B) Peak intensities of ^{13}C-methyl-oleate at different concentrations. C) Standard curve of ^{13}C-methyl-oleate intensity versus concentration. D) Calculated mole ratio of Can f 1 and Fel d 7 to the ^{13}C-methyl oleate detected in the sample.

Supplementary Table 1 Lipocalin Allergens[a]

**Animal**	**Lipocalin**
Dog	Can f 1[b, c], Can f 2, Can f 4, Can f 6[d]
Cat	Fel d 4[d], Fel d 7[c]
Cow	Bos d 2, Bos d 5
Guinea pig	Cav p 1[b], Cav p 2[b], Cav p 3, Cav p 6
Horse	Equ c 1[b], Equ c 2
Mouse	Mus m 1[b]
Rat	Rat n 1[b]
Rabbit	Ory c 1[b], Ory c 2[b], Ory c 4
Golden Hamster	Mes a 1
Siberian hamster	Phod s 1
American cockroach	Per a 4
German cockroach	Bla g 4
Pigeon Tick	Arg r 1[b]

[a] 24 out of 1051 allergens from http://www.allergen.org

[b] Major allergens

[c, d] Cross-reactive pair: Can f 1-Fel d 7, Can f 6-Fel d 4

Supplementary Table 2

Fatty Acids Screened[a]

Well	Product Name	Well	Product Name
A2	N-(α-Linolenoyl) Tyrosine	E2	Arachidic Acid
A3	N-Arachidonoyl-3-hydroxy-γ-Aminobutyric Acid	E3	Docosahexaenoic Acid ethyl ester
A4	N-Oleoyl-L-Serine	E4	Stearidonic Acid methyl ester
A5	Farnesyl Thiosalicylic Acid Amide	E5	N-Arachidonoyl-L-Serine
A6	Myriocin	E6	Dihomo-γ-Linolenic Acid methyl ester
A7	S-Farnesyl Thioacetic Acid	E7	Arachidonic Acid (sodium salt)
A8	cis-Parinaric Acid	E8	Lauric Acid
A9	Traumatic Acid	E9	Palmitic Acid
A10	Arachidonic Acid	E10	Oleic Acid-2,6-diisopropylanilide
A11	Arachidonic Acid methyl ester	E11	Stearidonic Acid ethyl ester
B2	N-Arachidonoyl-L-Alanine	F2	cis-7-Hexadecenoic Acid methyl ester
B3	N-Arachidonoyl-γ-Aminobutyric Acid	F3	Docosahexaenoic Acid methyl ester
B4	Eicosapentaenoic Acid	F4	cis-4,10,13,16-Docosatetraenoic Acid m[illegible]
B5	Eicosatetraynoic Acid	F5	cis-4,10,13,16-Docosatetraenoic Acid
B6	10(E),12(Z)-Conjugated Linoleic Acid	F6	cis-7-Hexadecenoic Acid
B7	Linoleic Acid	F7	Palmitic Acid methyl ester
B8	Linoelaidic Acid	F8	10-Thiastearic Acid
B9	Docosapentaenoic Acid	F9	Δ2-cis Eicosenoic Acid
B10	Docosatrienoic Acid	F10	Δ2-trans Eicosenoic Acid
B11	13(Z)-Docosenoic Acid	F11	CUDA
C2	11(Z),14(Z),17(Z)-Eicosatrienoic Acid	G2	AUDA
C3	α-Linolenic Acid	G3	Stearic Acid ethyl ester
C4	γ-Linolenic Acid	G4	Myristic Acid ethyl ester
C5	Dihomo-γ-Linolenic Acid	G5	Linoleic Acid ethyl ester
C6	Dihomo-γ-Linolenic Acid ethyl ester	G6	α-Linolenic Acid ethyl ester
C7	Elaidic Acid	G7	Arachidonic Acid ethyl ester
C8	Oleic Acid	G8	Oleic Acid ethyl ester
C9	17-Octadecynoic Acid	G9	Palmitic Acid ethyl ester
C10	MEDICA 16	G10	Lauric Acid ethyl ester
C11	Adrenic Acid	G11	Palmitoleic Acid ethyl ester
D2	Docosahexaenoic Acid	H2	9(Z),11(E),13(E)-Octadecatrienoic Acid [illegible]
D3	Stearidonic Acid	H3	9(Z),11(E),13(E)-Octadecatrienoic Acid [illegible]
D4	11(Z),14(Z)-Eicosadienoic Acid	H4	Myristoleic Acid methyl ester
D5	Phytanic Acid	H5	Pinolenic Acid
D6	9(E),11(E)-Conjugated Linoleic Acid	H6	Pinolenic Acid methyl ester
D7	2-fluoro Palmitic Acid	H7	Pinolenic Acid ethyl ester
D8	2-hydroxy Myristic Acid	H8	Ricinoleic Acid methyl ester
D9	8-methyl Nonanoic Acid	H9	Palmitoleic Acid
D10	Linoleoyl Glycine	H10	cis-12-Octadecenoic Acid methyl ester
D11	Docosahexaenoyl Glycine	H11	Stearic Acid

[a]Compound were purchased from Cayman chemicals.

Supplementary Table 3

Can f 1

Sample #	Ole	PIN	PH2	RAC
1	-46.8±0.4	-37.9±0.5	-54.3±0.5	-24.5±0.4
2	-48.1±0.4	-41.7±0.3	-44.8±0.6	-23.5±0.3
3	-44.2±0.4	-42.1±0.3	-39.4±0.4	-24.2±0.2
4	-43.7±0.3	-41.8±0.3	-43.0±0.5	-22.9±0.3
5	-42.4±0.4	-42.0±0.3	-44.0±0.4	-19.0±0.5

Fel d 7

Sample #	Ole	PIN	PH2	RAC
1	-35.8±0.3	-35.8±0.3	-46.6±0.5	-32.2±0.8
2	-37.3±0.3	-37.2±0.3	-47.6±0.5	-32.7±0.6
3	-45.9±0.6	-36.9±0.3	-44.1±0.6	-33.8±0.3
4	-44.6±0.5	-36.2±0.4	-43.1±0.4	-33.5±0.2
5	-45.1±0.5	-38.4±0.4	-50.1±0.4	-33.3±0.2
